# Sleep quality mediates the association between chronotype and mental health in young Indian adults

**DOI:** 10.1038/s44184-024-00076-9

**Published:** 2024-06-24

**Authors:** Satyam Chauhan, Rakesh Pandey, Krupa Vakani, Ray Norbury, Ulrich Ettinger, Veena Kumari

**Affiliations:** 1 Division of Psychology, Department of Life Sciences, College of Health, Medicine and Life Sciences, Brunel University London, Uxbridge, UK; 2 Centre for Cognitive and Clinical Neuroscience, College of Health, Medicine and Life Sciences, Brunel University London, Uxbridge, UK; 3 Department of Psychology, Faculty of Social Sciences, Banaras Hindu University, Varanasi, India; 4 Department of Psychology, University of Bonn, Bonn, Germany

**Keywords:** Neuroscience, Psychology, Risk factors, Signs and symptoms

## Abstract

There is increasing recognition of ‘higher preference for eveningness’ as a potential independent risk factor for poor mental health. To examine the chronotype-mental health relationship while also quantifying the potential roles of poor sleep quality, relevant personality traits, and childhood trauma, we assessed 282 young adults (18–40 years; 195 females) residing in North India, between January and March 2023 (to control for seasonal variation), using self-report measures of diurnal preference, sleep patterns, mental health (depression, anxiety, and stress), personality traits (extraversion, neuroticism, schizotypy, and impulsivity), and childhood trauma. The results showed a significant association between eveningness and poor mental health but this association was fully mediated by poor sleep quality. Neuroticism, emotional abuse and cognitive disorganisation were correlated with eveningness as well as with poor mental health and sleep quality. Neuroticism and emotional abuse, but not cognitive disorganisation, also had indirect effects on mental health via sleep quality. Our findings highlight the crucial role played by sleep quality in the chronotype-mental health relationship.

## Introduction

Chronotype is a multidimensional construct which reflects behavioural consequences and manifestations of various circadian mechanisms^1^. It is known to exist on a continuum between two extremes, i.e., early morning and late evening chronotype. Most individuals, however, fall in the middle of this continuum and are intermediate type. There is considerable evidence that the late chronotype (i.e., eveningness) is associated with adverse mental health outcomes, such as depression^2–5^, anxiety^6,7^, psychosis^8,9^, impulsive and maladaptive behaviour^10,11^, and substance abuse^12^. Furthermore, personality traits that are known to be associated with a higher risk of developing these mental disorders or problematic behaviours, namely, neuroticism (linked to depression and anxiety disorders^13^), schizotypy (psychosis^14^) and impulsivity (substance abuse^15^) also show a positive association with eveningness in non-clinical samples in some studies^1,16^. By contrast, extraversion which is associated with a lower risk for mental disorders^1,16,17^ may have a small association with the early chronotype (i.e., morningness)^18^.

There are reports that individuals with a higher preference for eveningness have poor quality or altered sleep patterns^19,20^, including spending less time in bed during weekdays, shorter sleep duration, daytime sleepiness and dysfunction, irregular sleep-wake cycles, and a need for more sleep on weekends^19,21–23^. Such self-reported sleep alterations are also common in various mental illnesses, for example major depression or anxiety disorders^24–27^. Furthermore, poor sleep quality has been consistently found in people with a history of childhood abuse/trauma^28–30^, and childhood maltreatment is an established risk factor for many disorders, including depression, anxiety, psychosis, personality disorder, post-traumatic stress disorder, and substance abuse^31–33^. Whether and to what extent a history of childhood trauma might influence any relationship between chronotype and mental health, however, remains unclear.

Against the backdrop of previous findings supporting a direct association between eveningness and poor mental health^2,6,7^, a study conducted in the UK^34^ reported a positive association between eveningness and depressive symptomatology and this association was partly mediated by subjective sleep quality. A more recent UK study by Muzni and colleagues^18^ involving a large non-clinical adult sample suggested a much stronger relationship between poor sleep quality and adverse mental health (with medium-to-large effect sizes), relative to that observed between eveningness and poor mental health (negligible-to-small effect sizes), especially in females. These findings raise doubts about the widely publicised association of eveningness with poor health outcomes, at least in the general population. There are, however, no data examining the chronotype-mental health relationship while also quantifying the influence of sleep quality, chronotype-relevant personality traits, and childhood trauma within the same homogenous sample of non-clinical adults.

The main aim of the present study, therefore, was to determine the association between chronotype and mental health (depression, anxiety, and stress) in young (18-40 years) males and females, with a particular focus on the roles of sleep quality, relevant personality traits (neuroticism, schizotypy, impulsiveness, and extraversion), and childhood trauma. We tentatively hypothesised, based on the findings of Muzni and colleagues^18^, that both eveningness and poor sleep quality would be associated with higher levels of depression, anxiety, and stress, with these associations being stronger for sleep quality than for eveningness. Lastly, we explored possible associations between chronotype, sleep quality, personality traits, childhood trauma and mental health, and examined the influence of personality traits and childhood trauma, while also considering sleep quality, in the chronotype-mental health relationship.

## Results

### Sample characterisation

The majority of the sample comprised of Asian Indians (92.2%), pursuing a bachelor’s degree or above (95.7%). Just over half (54.3%) of the sample self-reported consuming caffeine, and only 0.7% self-reported consuming alcohol (Table 1). About half of the sample (47.51) reported normal BMI, and a significant proportion (46.45%) reported being underweight (as also seen in other young North Indian cohorts^35^. Just over half of sample (55.3%) met the PSQI criteria for good sleep (score ≤ 5)

**Table 1:** Demographic characteristics of the study sample

Demographic characteristics	*N* = 282	Frequency (%) of *N* = 282
Ethnicity	Asian Indian	92.2%
	European White	0.8%
	Other Ethnic Groups	4.1%
	Prefer not to say	2.8%
Stimulant/Sedative Consumption	Caffeine	54.3%
	Nicotine	4.3%
	Alcohol	0.7%
	None	40.8%
Body Mass Index (BMI)^a^	Underweight (<18.5)	46.45%
	Normal (18.5–24.9)	47.51%
	Overweight (25–29.9)	4.25%
	Obese (>30)	0%
Education/Employment	Student	95.7%
	Full-time Work	4.3%
	Part-time Work	0%
Medication	Yes	0%
	No	92.6%
	Prefer not to say	7.4%
Sleep Quality^b^	Good (≤5)	55.3%
	Poor (6–15)	44.7%

^a^BMI data missing for five participants.

^b^Individuals scoring 5 or below on the PSQI were categorised as good sleepers and those scoring above 5 and below 15 (highest score in our sample) as poor sleepers.

Females were younger than males (*t*_*280*_ = 4.04, *p* < 0.001), had significantly lower BMI (*t*_*275*_ = 4.24, *p* < 0.001), had a higher morning preference (*t*_*280*_ = 4.53, *p* < 0.001), and rated themselves as having poorer sleep quality (*t*_*280*_ = 4.09, *p* < 0.001). They also scored higher than males on Neuroticism (*t*_*280*_ = 5.62, *p* < 0.001), Depression (*t*_*280*_ = 2.14, *p* = 0.033), Anxiety (*t*_*280*_ = 2.07, *p* = 0.039), Stress (*t*_*280*_ = 3.48, *p* < 0.001), Cognitive Disorganisation (*t*_*280*_ = 3.11, *p* = 0.002), Lack of Perseverance (*t*_*280*_ = 3.06, *p* = 0.002), Lack of Premeditation (*t*_*280*_ = 2.85, *p* = 0.005), and Emotional Abuse (*t*_280_ = 2.56, *p* = 0.011) (Table 2). Males had higher scores than females for Sensation Seeking (*t*_*280*_ = 7.30, *p* = 0.001), Positive Urgency (*t*_*280*_ = 3.84, *p* = 0.001), and Physical Neglect (*t*_*280*_ = 3.49, *p* = 0.001) (Table 2).

**Table 2:** Descriptive statistics for self-report measures (chronotype, mental health, sleep quality, personality traits and childhood trauma)

Study Variables			Males (*n* = 87)		Females (*n* = 195)		Entire Sample (*N* = 282)	
			Mean ± SD	Sample Range	Mean ± SD	Sample Range	Mean ± SD	Sample Range
Age			26.26 ± 4.80	18–40	23.98 ± 4.17	18–40	24.68 ± 4.49	18–40
Chronotype	MEQ		53.31 ± 9.05	31–77	47.98 ± 9.11	27–70	49.63 ± 9.40	27–77
		Depression	11.90 ± 10.24	0–42	14.98 ± 11.50	0–42	14.03 ± 11.20	0–42
Mental Health	DASS-21	Anxiety	11.60 + 9.33	0–42	14.28 ± 10.32	0–42	13.46 ± 10.08	0–42
		Stress	12.20 ± 8.59	0–42	16.25 ± 9.18	0–42	15.00 ± 9.18	0–42
		Sleep Quality	0.91 ± 0.66	0–3	1.22 ± 0.78	0–3	1.13 ± 0.76	0–3
		Sleep Latency	0.80 ± 0.69	0–2	1.02 ± 0.71	0–2	0.95 ± 0.71	0–2
		Sleep Duration	1.03 ± 0.78	0–3	1.03 ± 0.83	0–3	1.03 ± 0.81	0–3
Sleep Quality	PSQI	Sleep Efficiency	0.66 ± 1.01	0–3	0.80 ± 1.02	0–3	0.76 ± 1.02	0–3
		Sleep Disturbance	1.09 ± 0.49	0–3	1.29 ± 0.53	0–3	1.23 ± 0.53	0–3
		Sleep Medication	0.05 ± 0.23	0–1	0.22 ± 0.67	0–3	0.17 ± 0.58	0–3
		Daytime Dysfunction	0.75 ± 0.80	0–3	1.2 ± 0.85	0–3	1.06 ± 0.86	0–3
		Global Score	4.67 ± 2.31	0–13	6.02 ± 2.63	1–15	5.60 ± 2.61	0–15
	EPQ-S	Extraversion	7.28 ± 3.02	0–12	6.56 ± 3.52	0–12	6.79 ± 3.39	0–12
		Neuroticism	5.47 ± 3.23	0–12	7.80 ± 3.20	0–12	7.08 ± 3.38	0–12
		Unusual Experience	5.34 ± 3.172	0–12	5.43 ± 2.89	0–12	5.40 ± 2.97	0–12
		Cognitive Disorganisation	4.95 ± 3.16	0–11	6.23 ± 3.20	0–11	5.84 ± 3.24	0–11
		Introvertive Anhedonia	3.48 ± 1.80	0–8	3.66 ± 1.93	0–9	3.60 ± 1.89	0–9
Personality Traits	sO-LIFE	Impulsive Nonconformity	3.56 ± 2.03	0–9	3.54 ± 2.12	0–9	3.54 ± 2.09	0–9
		sO–LIFE Total	17.34 ± 8.05	1–36	18.87 ± 7.71	3–36	18.40 ± 7.84	1–36
		Negative Urgency	10.49 ± 2.98	4–16	9.98 ± 2.97	4–16	10.14 ± 2.98	4–16
		Lack of Perseverance	6.72 ± 1.87	4–11	7.52 ± 2.10	4–14	7.28 ± 2.06	4–14
	S-UPPS-P	Lack of Premeditation	6.27 ± 1.95	4–11	7.08 ± 2.28	4–14	6.83 ± 2.21	4–14
		Sensation Seeking	12.74 ± 2.16	7–16	10.43 ± 2.70	4–16	11.14 ± 2.76	4–16
		Positive Urgency	10.26 ± 3.21	4–16	8.75 ± 2.95	4–16	9.22 ± 3.10	4–16
		Emotional Abuse	9.56 ± 4.26	5–25	11.15 ± 5.031	5–25	10.66 ± 4.85	5–25
		Physical Abuse	8.49 ± 4.24	5–25	8.14 ± 4.58	5–25	8.25 ± 4.47	5–25
Childhood Trauma	CTQ-SF	Sexual Abuse	8.13 ± 4.31	5–21	9.04 ± 5.20	5–25	8.76 ± 4.95	5–25
		Emotional Neglect	11.55 ± 4.26	5–21	11.98 ± 4.97	5–25	11.85 ± 4.76	5–25
		Physical Neglect	9.85 ± 3.27	5–17	8.35 ± 3.22	5–19	8.81 ± 3.30	5–19
		CTQ Total	47.59 ± 16.37	25–100	48.68 ± 17.49	25–110	48.35 ± 17.13	25–110

*MEQ* Morningness-Eveningness Questionnaire, *PSQI* Pittsburgh Sleep Quality Index, *EPQ-SF* Eysenck Personality Questionnaire-Revised, *DASS-21* Depression Anxiety and Stress Scale-21 Items, *sO-OLIFE* short Oxford-Liverpool Inventory of Feelings and Emotions, *S-UPPS-P* Impulsive Behaviour Scale-Short Version, *CTQ-SF* Short Form of Childhood Trauma Questionnaire.

### Chronotype, sleep quality, mental health, personality traits and childhood trauma

Higher preference for eveningness (i.e., lower MEQ scores) correlated with higher scores on Depression (*r* = −0.308*, p* = 0.001), Anxiety (*r* = −0.213*, p* = 0.001), Stress (*r* = −0.267*, p* = 0.001) scales; higher scores on personality measures of Neuroticism (*r* = −0.299*, p* = 0.001), Cognitive Disorganisation (*r* = −0.287*, p* = 0.001), Lack of Perseverance (*r* = −0.181, *p* = 0.002), Lack of Premeditation (*r* = −0.180, *p* = 0.002), and Sensation Seeking (*r* = −0.215, *p* = 0.001); as well as Emotional Abuse (*r* = −0.196, *p* = 0.001) and Emotional Neglect (*r* = −0.153, *p* = 0.001) (Table 3). Higher preference for morningness was associated with higher Extraversion scores (*r* = 0.222*, p* = 0.001) (Table 3). Of these correlations, the correlation between eveningness and Lack of Premeditation appeared stronger in males than females (Fisher’s Exact z = 2.01, *p* = 0.044) though this sex difference failed to maintain statistical significance after Bonferroni correction for multiple comparisons (*p* > 0.0025). BMI did not correlate significantly with chronotype, any personality traits, sleep quality, and mental health.

**Table 3:** Correlations (Pearson’s *r*) between chronotype and measures of mental health, sleep quality, personality traits and childhood trauma

	Mental Health			Sleep Quality	Personality Traits											Childhood Trauma				
	DASS-21			PSQI	EPQ-SF		sO-LIFE				S-UPPS-P					CTQ-SF				
	D	A	S		E	N	UE	CD	IA	IN	NU	LP	LPr	SS	PU	EA	PA	SA	EN	PN
MEQ (Overall)	−0.308 (0.001)	−0.213 (0.001)	−0.267 (0.001)	−0.389 (0.001)	0.299 (0.001)	−0.222 (0.001)	−0.078 (0.194)	−0.287 (0.001)	−0.086 (0.152)	−0.084 (0.157)	−0.067 (0.265)	−0.181 (0.002)	−0.180 (0.002)	0.215 (0.001)	0.079 (0.186)	−0.196 (0.001)	0.034 (0.567)	0.073 (0.219)	−0.153 (0.001)	0.006 (0.923)
Males	−0.204 (0.057)	−0.057 (0.597)	−0.111 (0.307)	−0.163 (0.132)	0.274 (0.01)	−0.199 (0.065)	−0.103 (0.341)	−0.267 (0.012)	−0.103 (0.343)	−0.017 (0.879)	−0.028 (0.796)	−0.288 (0.007)	−0.326 (0.002)	0.198 (0.066)	0.025 (0.817)	−0.193 (0.073)	0.012 (0.912)	0.062 (0.566)	−0.093 (0.393)	−0.106 (0.328)
Females	−0.320 (0.001)	−0.241 (0.001)	−0.274 (0.001)	−0.421 (0.001)	0.179 (0.012)	−0.252 (0.001)	−0.064 (0.376)	−0.245 (0.001)	−0.066 (0.357)	−0.119 (0.097)	−0.118 (0.099)	−0.084 (0.245)	−0.075 (0.299)	0.103 (0.151)	0.02 (0.781)	−0.153 (0.033)	0.032 (0.662)	0.113 (0.114)	−0.168 (0.019)	−0.028 (0.702)

*MEQ* Morningness-Eveningness Questionnaire, *D* Depression, *A* Anxiety, *S* Stress, *DASS-21* Depression Anxiety and Stress Scale-21 Items, *PSQI* Pittsburgh Sleep Quality Index, *E* Extraversion, *N* Neuroticism, *EPQ-SF* Eysenck Personality Questionnaire-Revised, *UE* Unusual Experience, *CD* Cognitive Disorganisation, *IA* Introvertive Anhedonia, *IN* Impulsive Nonconformity, *sO-LIFE* short Oxford-Liverpool Inventory of Feelings and Emotions, *NU* Negative Urgency, *LP* Lack of Perseverance, *LPr* Lack of Premeditation, *SS* Sensation Seeking, *PU* Positive Urgency, *S-UPPS-P* Impulsive Behaviour Scale-Short Version, *EA* Emotional Abuse, *PA* Physical Abuse, *SA* Sexual Abuse, *EN* Emotional Neglect, *PN* Physical Neglect, *CTQ-SF* short form of Childhood Trauma Questionnaire.

Poor sleep quality (i.e., higher PSQI scores) correlated with higher levels of Depression (*r* = 0.489*, p* < 0.001), Anxiety (*r* = 0.474*, p* < 0.001), Stress (*r* = 0.518*, p* < 0.001); higher scores on personality measures of Neuroticism ( = 0.433*, p* < 0.001), Unusual Experiences (*r* = 0.168*, p* = 0.001), Cognitive Disorganisation (*r* = 0.294*, p* = 0.001), Introvertive Anhedonia (*r* = 0.150*, p* = 0.012), Impulsive Nonconformity (*r* = 0.198*, p* = 0.001), and Negative Urgency (*r* = 0.141*, p* = 0.017); and severity of self-reported Emotional Abuse (*r* = 0.377*, p* = 0.001), Emotional Neglect (*r* = 0.275*, p* = 0.001), and Physical Abuse (*r* = 0.164*, p* = 0.006). Poor sleep also correlated with lower scores on Extraversion (*r* = −0.125*, p* < 0.001). Poor sleep quality correlated with eveningness (*r* = −0.389*, p* < 0.001); although this correlation appeared stronger in females than males (Fisher’s Exact *z* = 2.17, *p* = 0.029), this sex difference did not survive correction for multiple comparisons (*p* > 0.0025). Lastly, compared to eveningness, poor sleep quality showed significantly stronger correlations, as expected, with Depression (Fisher’s Exact *z* = 2.55, *p* = 0.01), Anxiety (Fisher’s Exact *z* = 3.53, *p* < 0.001), and Stress (Fisher’s Exact z = 3.54, *p* < 0.001).

### The mediating role of sleep quality in chronotype mental health relationship

**Fig. 1:**
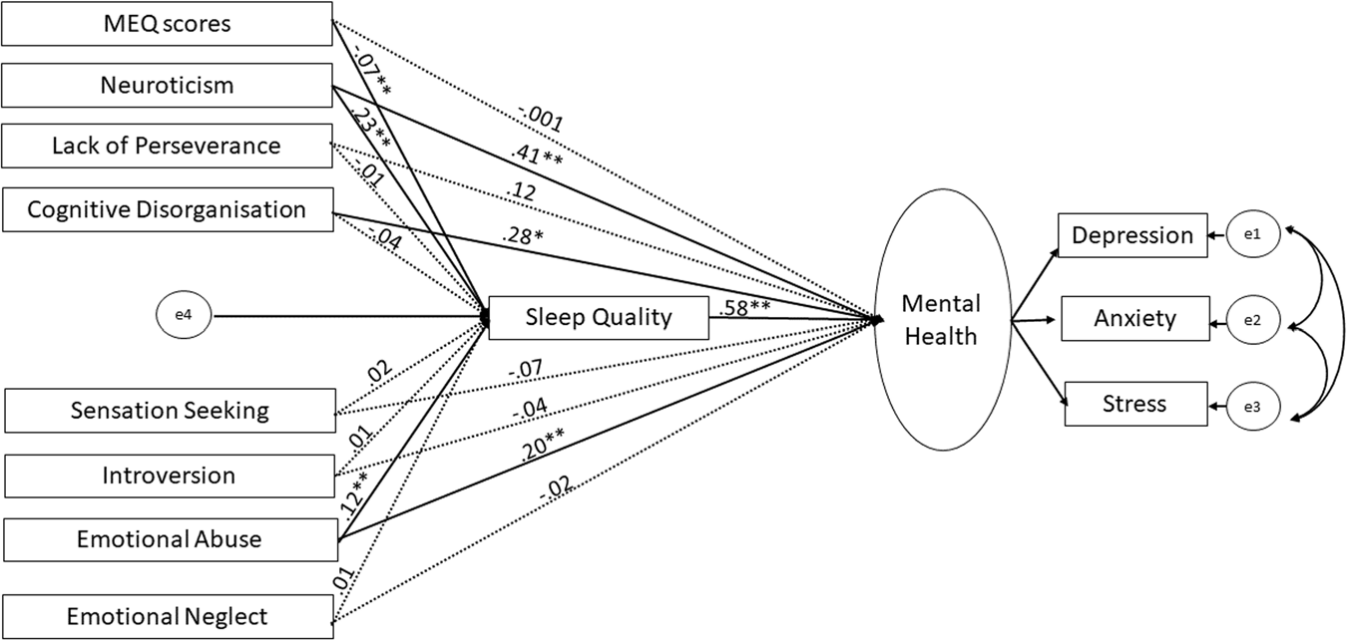
Results of the SEM analyses, with solid lines representing significant paths (**p < 0.001, *p < 0.005) and dotted lines representing non-significant paths.

**Fig. 2:**
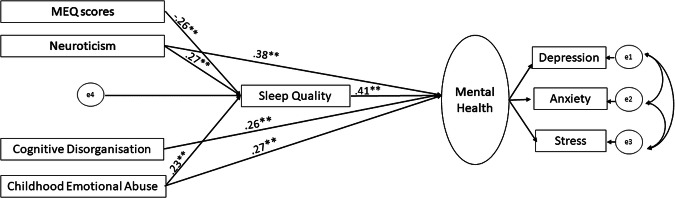
Results of the SEM analyses for the revised model showing all significant paths (** < 0.001).

Our initial model (see Statistical analysis) was found to be a very good fit [(χ2/*df* = 2.11, *p* < 0.001), RMSEA (.06), GFI (.97), AGFI (.90), and CFI (.98)] to the data, though some direct effects (paths) were non-significant (see Fig. 1). The model was, therefore, revised because of poor local fit (i.e., presence of nonsignificant path coefficients) by removing the non-significant paths leaving us with the final model [model fit indices: χ2/*df* = 1.18; GFI = 0.98; TLI = 0.99; CFI = 0.99; RMSEA = 0.02)] (Fig. 2). As shown in Fig. 2, we did not find any direct effect of eveningness (MEQ scores) on mental health (β = −0.001, *p* = 0.961), and found its relationship with (poor) mental health to be fully mediated by poor sleep quality (β = −0.10, *p* < 0.001). Poor sleep quality also partially mediated the relationship between childhood emotional abuse and poor mental health (*β* = 0.96, *p* < 0.001), and between higher neuroticism and poor mental health (*β* = 0.11, *p* < 0.001), but not between Cognitive Disorganisation and poor mental health (*β* = −0.04, *p* = 0.427). While exploring sex differences, we observed that the comparison of model fit of unconstrained and fully constrained model revealed a non-significant chi-square difference Δ*χ*^2^(20) = 25.87, *p* = 0.156 and a non-significant difference in CFI (ΔCFI = 0.005), suggesting the model to be invariant in males and females. However, the difference in RMSEA was found to be higher than the prescribed cut-off of .01 (ΔRSMEA = 0.02) suggesting non-invariance. Therefore, we tested pairwise difference in the path coefficients of unconstrained model in males and females and found a significant difference (stronger in females) in the direct path linking Cognitive Disorganization to mental health (Critical ratio=2.138, *p* < 0.05).

## Discussion

This is the first study, to our knowledge, to investigate chronotype-mental health associations while also examining the roles of sleep quality, clinically relevant personality traits and childhood trauma in this association. The main findings were: (i) a preference for eveningness had small-to-medium correlations (*r* values: 0.20–0.30) while poor sleep quality had medium-to-large correlations (*r* values: 0.47–0.52) with mental health outcomes (depression, anxiety, and stress), (ii) eveningness had significant but mostly small-sized (*r* values > 0.30) associations with various personality traits and self-reported history of childhood emotional abuse and neglect, and (iii) there was no significant direct effect of eveningness on mental health outcomes, with sleep quality fully mediating the chronotype-mental health relationship. Although, on average, females displayed more morningness than males, sex did not significantly influence any chronotype-mental health associations.

The findings in relation to our first hypothesis demonstrated only small-to-medium (at best) positive associations between a preference for eveningness and poor mental health outcomes (depression, anxiety, stress) in a North Indian, young and healthy volunteer sample, as also found in previous studies of general population samples in Western countries (UK^18^, *f2* = 0.024; Canada^5^, *ηp2* = 0.02–0.04). Our finding of relatively stronger (medium-to-large) positive associations between poor sleep quality and poor mental health outcomes, compared to those seen between eveningness and mental health outcomes, confirm our hypothesis, and offers further support to earlier findings in a non-clinical sample in the UK^18^. Furthermore, this study supports previous findings^1^ in showing significant but mostly small associations between eveningness and higher scores on measures of neuroticism^18,36^ and impulsivity^11,37,38^. Extraversion has also been found to have a small positive association with eveningness^36^, though not consistently^1,16^. Interestingly, our findings also revealed a significant positive association (*r* = 0.299) between eveningness and the cognitive disorganisation aspect of schizotypy with non-significant associations in the same direction for other aspects of schizotypy. In a previous study^39^ that did not find any relationship between eveningness and schizotypy, a positive association between all schizotypy (O-LIFE) dimensions and altered biological rhythms in patients with bipolar disorder and healthy controls was observed, and it was present most strongly for Cognitive Disorganisation, although the mechanisms underlying such an association, remain unclear at present.

The findings in relation to our second hypothesis demonstrated no direct effect of eveningness on mental health outcomes, and instead showed that eveningness-poor mental health relationship was fully mediated by poor sleep quality. It is well known that numerous environmental and social factors associated with modern-day lifestyles hinder regular sleep patterns^40,41^, and contribute to irregular secretion of melatonin which in turn has been linked to mental disorders, such as psychosis and major depression^42,43^. Not surprisingly, most individuals with an evening preference accumulate higher social jetlag, sleep pressure, and sleep deprivation^21,22,40^. Sleep disturbances affect the reactivity of neuroendocrine stress systems and responsivity, reducing the ability to cope with emotional dysfunction^44^. Chronic and acute sleep-related issues may fundamentally change the brain chemistry and neuroendocrine systems (e.g., altered hypothalamic pituitary adrenal axis^44^). Poor sleep may further sensitise people with high levels of neuroticism to experience negative affect and emotional (limbic) arousal^45^. Individuals with a history of emotional abuse are also reported to experience emotional dysfunction and distress, which in turn may contribute to poor sleep quality and altered circadian rhythms^46,47^ and elevate risk for affective and stress-related disorders^29,32,48^. In this context, it is noteworthy that emotional abuse appeared relatively more important than other types of abuse for mental health, as also argued in the context of prevalence of mental disorders in children with a history of physical abuse^33,49^. Individuals with high levels of schizotypy also often experience low mood^50^ and report social anxiety, distress as well as higher sensitivity to social rejections^51^, all of which contribute to poor mental health. Therefore, prolonged and/or acute poor sleep quality, neuroticism, history of emotional abuse, and schizotypy may explain why eveningness has been associated with poor mental health, though with a marked variation in effect sizes^4,52^ possibly due to its dependency on the quality of sleep.

The present study had a number of strengths. First, it used a homogenous sample of young English-speaking, healthy adults residing in North India, with <5% consuming nicotine and alcohol as self-reported. Second, all data were collected over a brief period to minimise any season-related influences. Third, chronotype was used as a continuous variable to preserve power. Our study also had a number of limitations. First, although we used validated self-report questionnaires with sensitivity ranging between 73 and 97.7%^53^, there were no objective markers of chronotype. This, however, may not be a serious concern given significant correlations and overlaps between subjective and objective chronotype measures even in a clinical sample^54^. Second, we did not collect information on natural and/or artificial light exposure that causes phase delay in circadian rhythms^40,55,56^. Third, our sample was predominantly female, limiting our ability to investigate sex differences adequately. Additionally, we did not collect information on cyclic fluctuation of reproductive hormones which may act as a potential confounding variable. Fourth, we did not examine the influence of socioeconomic status, family dynamics or cultural beliefs, which may also directly or indirectly influence an individuals’ mental health. Fifth, our findings from a young North Indian sample may or may not generalise to non-Indian or older age-groups. Lastly, as this was a correlational study, the findings cannot conclusively speak to causation. Despite these limitations, our findings might still have important implications. Specifically, we speculate that personal and societal interventions aiming to promote good sleep, especially in high-risk groups (e.g., with high neuroticism or emotional abuse), may help to promote good mental health in the general population.

In conclusion, this study found no direct relationship between a preference for eveningness and poor mental health outcomes (depression, anxiety, and stress) in young adults. Instead, this relationship was mediated by poor sleep quality. Our findings argue against ‘eveningness’ as an independent risk factor for poor mental health, and indirectly suggest that promotion of good quality sleep may provide a more helpful strategy than those aiming to shift diurnal preferences towards morningness for improving mental health, especially in high-risk groups. Further studies in other cultures, settings, age groups, and using direct measures of circadian (mis)alignments and sleep quality along with self-report measures to collect data on more than one occasion within the same individuals, are needed to examine the stability and generalisability of these findings and realise their full potential for promoting mental health in the general population.

## Methods

### Participants and design

The data were collected from young adults (*N* = 313, age range 18–40 years) residing in different parts of Northern India between January and March 2023 with average daytime temperatures ranging between 14 and 23 °C. The age range was restricted to 18–40 years given previous evidence of age-related changes in chronotype^57^.

Of 313, 31 participants had to be excluded for failing our attention check criteria (i.e., failed to enter a given response to one or more of the four catch items; *n* = 20) or due to non-completion of some measures (*n* = 11), leaving a final sample of 282 participants (195 females, 87 males) who completed all self-report measures online in a single session. The inclusion criteria required all participants to be aged between 18–40 years and living in India, be fluent in English, not be on any regular medication, not have a history of any diagnosed mental disorders or drug abuse (any past or current use of non-prescribed drugs), and be able to provide written informed consent. Their participation was voluntary, and no compensation was provided. This study was approved by the Research Ethics Committee, College of Health, Medicine, and Life Sciences, Brunel University London (ref no. 41125-MHR-Mar/2023- 44225-4).

### Self-report measures

The *Morningness-Eveningness Questionnaire* (MEQ)^58^, which is a self-report questionnaire comprising of 19 items, was used to assess diurnal preference. The questionnaire has both a Likert scale (e.g., *item 19: are you a morning or evening type?*) and time scale (e.g., *item 18: at approximately what time of the day do you usually feel your best?)*. Twelve items on the Likert scale present four options, with the lowest values indicating definite eveningness. The remaining seven items on the time scale are divided into periods of 15 min, spanning a time frame of seven hours. Higher scores indicate a preference for morningness and lower scores indicate a preference for eveningness. The scale has high internal consistency (*a* = 0.83^58^; *a* = 0.76 in the current sample).

Depression, anxiety, and stress levels were assessed using the 21-item *Depression, Anxiety and Stress Scale* (DASS-21)^59^. It has three sub-scales: Depression, Anxiety, and Stress. Each sub-scale consists of seven items. The participants respond to each item based on their feelings on most days over the past week. The Depression scale assesses dysphoria, hopelessness, devaluation of life, self-deprecation, lack of interest, anhedonia, and inertia. The Anxiety scale assesses autonomic arousal, skeletal muscle effects, situational anxiety, and subjective experience of anxious affect. The Stress scale assesses difficulty relaxing, nervous arousal, getting easily upset, agitated, irritable, over-reactive, and impatient. The sub-scales are reported to have high reliability coefficients [Depression (*a* = 0.83–0.94), Anxiety (*a* = 0.66–0.87), Stress (*a* = 0.79–0.91)]^60^. The Cronbach’s alphas for Depression (*a* = 0.88), Anxiety (*a* = 0.84), and Stress (*a* = 0.81) sub-scales also indicated good internal consistency in the current sample.

Sleep quality was assessed using the *Pittsburgh Sleep Quality Index* (PSQI)^61^. The PSQI is a 19-item scale assessing daytime dysfunction, use of sleeping medication, sleep disturbances, habitual sleep efficiency, sleep duration, sleep latency, and subjective sleep score. Participants answer the PSQI questions for each of these components by relating them to their past month. Each component is scored from “No difficulty” (0) to “Severe difficulty” (3) and tallied up to yield a total score (range 0–21). Higher scores indicate poor sleep quality. The PSQI (global score) is reported to have a high internal consistency (*a* = 0.83) and test-retest reliability (*r* = 0.85), with a sensitivity of 89.6% and a specificity of 86.5%^61^. Cronbach’s alpha for the PSQI (global score) in the current sample was 0.67.

Extraversion, Neuroticism, and Psychoticism were measured using the short 48-item *Eysenck Personality Questionnaire-Revised* (EPQR-S)^62^. It has three sub-scales, corresponding to the three personality dimensions in the Eysenck’s model of personality, plus a lie scale^62^. Each scale contains 12 items with a binary response, ‘Yes’ or ‘No’ (scored as 1 or 0). Extraversion (*a* = 0.74–0.84) and Neuroticism scales (*a* = 0.70–0.77) are known to have good reliability but the Psychoticism scale is reported to have less-than-satisfactory reliability (*a* = 0.33–0.52^63^; as was also the case in the current sample (Extraversion, *α* = 0.82; Neuroticism, *α* = 0.82; Psychoticism, *α* = 0.27).

Schizotypal personality traits were assessed using the short version of the *Oxford-Liverpool Inventory of Feelings and Emotions* (sO-LIFE)^64,65^. It is a 43-item questionnaire with high reliability (*a* = 0.78–0.87) as well as good convergent and discriminant validity^66^. Each item belongs to one of the four sub-scales: (i) Unusual Experiences (12 items; describing perceptual aberrations, magical thinking, and hallucinations), (ii) Cognitive Disorganization (11 items; covering aspects of poor attention, concentration, decision-making, and social anxiety), (iii) Introvertive Anhedonia (10 items; describing a lack of enjoyment from social and physical sources of pleasure as well as avoidance of intimacy), and (iv) Impulsive Non-conformity (10 items; describing impulsive, anti-social, and eccentric forms of behaviour, often suggesting a lack of self-control). All items require a Yes/No response (scored 1 or 0). Higher scores indicate higher levels of schizotypy. Cronbach’s alphas in the current sample for Unusual Experiences, Cognitive Disorganisation, Introvertive Anhedonia, and Impulsive Nonconformity were 0.75, 0.81, 0.44, and 0.54, respectively.

Impulsivity was assessed using the *Impulsive Behaviour Scale*-*Short Version* (S-UPPS-P)^67^. It is a 20-item self-report measure with adequate reliability (*a* = 0.74–0.88)^67^. Each item is rated on a four-point Likert scale [(1) disagree strongly, (2) disagree some, (3) agree some, and (4) agree strongly]. There are five (5-item) sub-scales: Lack of Perseverance (inability to stay focused on a task), Lack of Premeditation (inability to account to the repercussions of actions), Sensation Seeking (tendency to seek unique and exciting experiences), Negative Urgency (tendency to react rashly in an intense negative mood), and Positive Urgency (tendency to react rashly in an intense positive mood). Higher scores indicate higher levels of impulsivity. Cronbach’s alphas in the current sample for Negative Urgency, Lack of Perseverance, Lack of Premeditation, Sensation Seeking, Positive Urgency were 0.73, 0.55, 0.73, 0.66, and 0.78, respectively.

Childhood trauma was assessed using the short form of the *Childhood Trauma Questionnaire* (CTQ-SF)^68^. It consists of 28 items on histories of abuse and neglect. It has five 5-item sub-scales, measuring emotional, physical, and sexual abuse, and emotional and physical neglect. All items are rated from ‘never true’ (score 1) to ‘very often true’ (score 5), and after reversing seven items, the scores on all sub-scales can range between 5 and 25. The final scores are classified as ‘none to minimal’, ‘low to moderate’, ‘moderate to severe’, and ‘severe to extreme’. Three additional items compose the minimisation/denial sub-scale for detecting socially desirable responses or false-negative trauma reports. The total CTQ score reflects the severity of multiple forms of abuse and neglect. These sub-scales are reported to have high test-retest reliability (*α* = 0.79–0.86) and internal consistency (*α* = 0.66–0.92^68^), though in the current sample, Cronbach’s alphas for Emotional Abuse, Physical Abuse, Sexual Abuse, Emotional Neglect, and Physical Neglect were 0.81, 0.87, 0.89, 0.82, and 0.58, respectively.

### Statistical analysis

Data were analysed using Statistical Package for Social Sciences (SPSS, for macOS, version 28; IBM, New York, United States), unless specified otherwise. Alpha level for testing the significance of effects was maintained at *p* < 0.05, unless stated.

The data on all self-report measures were examined and found to be suitable (skewness and kurtosis <±2) for parametric statistical approaches. The Psychoticism sub-scale of the EPQR-S was excluded from all analysis due to its low reliability in the current sample (*α* = 0.27) (a common problem with this sub-scale, as mentioned earlier). We explored possible sex differences in various self-report measures using independent sample *t*-tests. Since we considered chronotype as a continuous variable, Pearson’s *r* was used to examine the associations of chronotype (morningness-eveningness) with mental health (Depression, Anxiety, Stress), personality traits (Extraversion, Neuroticism, Schizotypy, and Impulsivity), and childhood trauma scores, followed by Fisher’s Exact *z*-test to test for significant differences in chronotype-mental health and sleep-mental health correlations as well as any sex differences in these correlations. Effect sizes for correlation coefficients were interpreted based on Cohen^69^ (*r* value ±0.1 to ±0.29 as small, ±0.3 to ±0.49 as medium, and ±0.5 to ±1 as large).

**Fig. 3:**
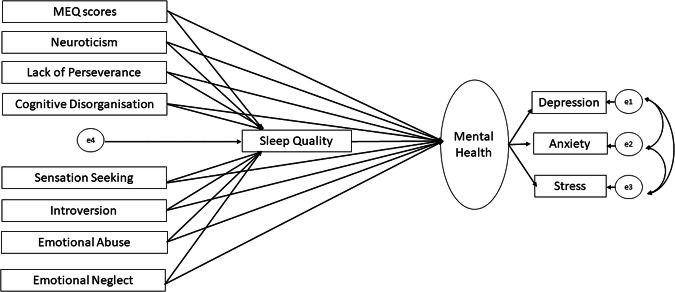
Schematic representation of proposed structural model showing the direct and indirect effects (via sleep quality) of chronotype (higher MEQ scores represent a greater preference for morningness and lower scores a greater preference for eveningness), personality traits, and childhood trauma (predictors) and mental health (outcome) relationship using SEM framework. All predictors were allowed to covary. The latent variable is indicated in an oval shape, observable variables in a rectangle shape, and e1, e2, e3, and e4 are residuals.

Given significant associations of eveningness with mental health, sleep quality, certain personality traits (Extraversion, Neuroticism, Cognitive Disorganisation, Lack of Perseverance, Lack of Premeditation, Sensation Seeking), and childhood emotional abuse and neglect (see Results), we conducted structural equation modelling (SEM) in SPSS AMOS with scores on the MEQ, personality traits, childhood emotional abuse and neglect as predictors, PSQI scores (sleep quality) as a mediator, and mental health (a latent construct, incorporating depression, anxiety, and stress) as the outcome variable (Fig. 3). All predictor, mediator, and outcome variables were checked for multicollinearity, with no significant violation (variance inflation factor <5, tolerance >0.2) found in the measured cases. The predictors were allowed to covary in the proposed model (see Fig. 3) and the maximum likelihood method was used to test the model fit and calculate the parameter estimates of path coefficients. We used the comparative fit index (CFI; >0.95 represents good model fit), root mean square of approximation (RMSEA; value >0.80 represents good fit), the ratio of maximum-likelihood chi-square to the degree of freedom (*χ*^2^/*df;* acceptable value < 5), the goodness of fit index (GFI; acceptable value >0.95), Tucker Lewis Index (TLI; >0.95), and adjusted goodness of fit index (AGFI, acceptable value >0.90) to evaluate the global fit of the model^70^. A global fitting model may have local misfit (i.e., presence of nonsignificant direct/indirect effects), therefore, the statistical significance of the indirect and direct effects was tested based on bias-corrected 95% bootstrap confidence intervals and associated *p* values. The model was then revised to exclude non-significant paths (Fig. 1; see Results) one-by-one leaving us with the significant direct or indirect paths in the final model (Fig. 1). The invariance of the model was inferred if the fully constrained model (measurement weights of measurement model of mental health as well as the structural weights, covariances and residuals were constrained to be equal in males and females) did not differ significantly from the unconstrained model. A non-significant chi-square difference (Δ*χ*^2^ with *p* > 0.05), ΔCFI ≤ 0.005, and ΔRMSE ≤ 0.01 is considered as evidence for invariance of a given model^71,72^.

### Supplementary information

Supplementary information

## Data Availability

All data supporting this work are freely available via Brunel University London research repository at 10.17633/rd.brunel.25451407.
