# Supplementary material for: Sleep quality mediates the association between chronotype and mental health in young Indian adults
Source: Npj Ment Health Res. 2024 Jun 24;3:31. doi: 10.1038/s44184-024-00076-9 (PMC11196584; doi:10.1038/s44184-024-00076-9)
Supplement: Supplementary file 1 — Supplementary information [file 44184_2024_76_MOESM1_ESM.pdf]

Supplementary Table 1. Correlations (Pearson's *r*) amongst measures of mental health, sleep quality, personality traits and childhood trauma

	Mental Health			Sleep Quality	Personality Traits											Childhood Trauma				
	DASS-21				EPQ-SF		sO-OLIFE				S-UPPS-P					CTQ-SF				
	D	A	S	PSQI	E	N	UE	CD	IA	IN	NU	LP	LPr	SS	PU	EA	PA	SA	EN	PN
D	1	.704 (.001)	.758 (.001)	.489 (.001)	-.261 (.001)	.526 (.001)	.288 (.001)	.496 (.001)	.305 (.001)	.376 (.001)	.324 (.001)	.236 (.001)	.325 (.001)	-.204 (.001)	.169 (.004)	.457 (.001)	.230 (.001)	.141 (.018)	.374 (.001)	.262 (.001)
A		1	.743 (.001)	.474 (.001)	-.133 (.026)	.461 (.001)	.380 (.001)	.369 (.001)	.291 (.001)	.368 (.001)	.268 (.001)	.065 (.277)	.193 (.001)	-.152 (.011)	.208 (.001)	.371 (.001)	.238 (.001)	.179 (.003)	.250 (.001)	.214 (.001)
S			1	.518 (.001)	-.169 (.004)	.562 (.001)	.277 (.001)	.464 (.001)	.252 (.001)	.342 (.001)	.301 (.001)	.112 (.061)	.234 (.001)	-.234 (.001)	.135 (.023)	.418 (.001)	.207 (.001)	.197 (.001)	.280 (.001)	.118 (.048)
PSQI				1	-.125 (.036)	.433 (.001)	.168 .005	.294 (.001)	.150 (.012)	.198 (.001)	.141 (.017)	.08 (.181)	.160 (.007)	-.145 0.015	.069 (.25)	.377 (.001)	.164 (.006)	.092 (.125)	.275 (.001)	.049 (.414)
E					1	-.248 (.001)	-.025 (.671)	-.283 (.001)	-.486 (.001)	.009 (.877)	-.058 (.33)	-.235 (.001)	-.124 (.038)	.334 (.001)	.1 (.094)	-.111 (.063)	.01 (.869)	.069 (.245)	-.217 (.001)	.076 (.206)
N						1	.355 (.001)	.609 (.001)	.311 (.001)	.358 (.001)	.428 (.001)	.053 (.375)	.238 (.001)	-.237 (.001)	.247 (.001)	.342 (.001)	.123 (.039)	.121 (.043)	.345 (.001)	.142 (.017)
UE							1	.530 (.001)	.298 (.001)	.523 (.001)	.340 (.001)	.072 (.231)	.159 (.008)	.061 (.31)	.451 (.001)	.324 (.001)	.282 (.001)	.201 (.001)	.314 (.001)	.358 (.001)
CD								1	.370 (.001)	.518 (.001)	.442 (.001)	.250 (.001)	.349 (.001)	-.117 (.05)	.362 (.001)	.353 (.001)	.199 (.001)	.136 (.022)	.408 (.001)	.254 (.001)
IA									1	.306 (.001)	.286 (.001)	.212 (.001)	.214 (.001)	-.193 (.001)	.182 (.002)	.288 (.001)	.225 (.001)	.126 (.034)	.381 (.001)	.213 (.001)
IN										1	.425 (.001)	(.075) (.212)	.235 (.001)	(.085) (.154)	.463 (.001)	.363 (.001)	.295 (.001)	.206 (.001)	.414 (.001)	.363 (.001)
NU											1	-.141 (.018)	.075 (.209)	.134 (.024)	.649 (.001)	.182 (.002)	.249 (.001)	.151 (.011)	.345 (.001)	.294 (.001)
LP												1	.535 (.001)	-.191 (.001)	-.135 (.023)	.190 (.001)	.1 (.094)	.087 (.147)	.087 (.143)	.119 (.046)
LPr													1	-.188 (.002)	.123 (.039)	.229 (.001)	.205 (.001)	.136 (.023)	.141 (.018)	.180 (.002)
SS														1	.318 (.001)	-.234 (.001)	-.05 (.401)	-.077 (.196)	-.075 (.207)	.098 (.101)
PU															1	.108 (.071)	.251 (.001)	.143 (.016)	.297 (.001)	.394 (.001)
EA																1	.656 (.001)	.520 (.001)	.570 (.001)	.416 (.001)
PA																	1	.562 (.001)	.391 (.001)	.499 (.001)
SA																		1	.278 (.001)	.378 (.001)
EN																			1	.529 (.001)
PN																				1

Abbreviations**:** D, Depression; A, Anxiety; S, Stress; DASS-21, Depression Anxiety and Stress Scale-21 Items; PSQI**,** Pittsburgh Sleep Quality Index ; E, Extraversion; N, Neuroticism; EPQ-SF, Eysenck Personality Questionnaire-Revised; UE, Unusual Experience; CD, Cognitive Disorganisation; IA, Introvertive Anhedonia; IN, Impulsive Nonconformity; sO-OLIFE, Oxford-Liverpool Inventory of Feelings and Emotions- Short Form; NU, Negative Urgency; LP, Lack of Perseverance; LPr, Lack of Premeditation; SS, Sensation Seeking;

PU, Positive Urgency; S-UPPS-P, Impulsive Behaviour Scale-Short Version; EA, Emotional Abuse; PA, Physical Abuse; SA, Sexual Abuse; EN, Emotional Neglect; PN, Physical Neglect; CTQ-SF, Childhood Trauma Questionnaire-Short Form.
